# The “Plan-Do-Check-Action” Plan Helps Improve the Quality of the “Standardized Training of Resident Physicians”: An Analysis of the Results of the First Pass Rate

**DOI:** 10.3389/fpubh.2020.598774

**Published:** 2021-02-23

**Authors:** Biyun Tang, Danfeng Lin, Fengjiang Zhang, Mengling Yan, Anwen Shao

**Affiliations:** ^1^Clinical Skills Training Center, The Second Affiliated Hospital, School of Medicine, Zhejiang University, Hangzhou, China; ^2^Department of Surgical Oncology, The Second Affiliated Hospital, School of Medicine, Zhejiang University, Hangzhou, China; ^3^Department of Anesthesiology, The Second Affiliated Hospital, School of Medicine, Zhejiang University, Hangzhou, China; ^4^Department of Neurosurgery, The Second Affiliated Hospital, School of Medicine, Zhejiang University, Hangzhou, China

**Keywords:** the standardized training of resident physicians, assessment results, PDCA plan, first pass rate, education

## Abstract

**Background:** Medical education is a demanding lifelong learning process, which includes three tightly connected stages: college education, post-graduate education, and continuous education. Residency, the first several years after a college education, is a pivotal time in the development of a qualified doctor. Additionally, residents are the main force that undertakes much of the clinical work in hospitals. Therefore, guaranteeing and improving residents' clinical skills and abilities through the standardized training of resident physicians (STRP) is important. However, compared with other hospitals in the Zhejiang Province, the STRP assessment results of residents in our hospital were not satisfactory in recent years. Therefore, the objective of this study was to find the problems causing the unsatisfactory performance and identify the role of the “Plan-Do-Check-Action” (PDCA) plan in providing a valuable framework for future training.

**Methods:** Relevant studies of STRP in China and abroad were investigated by the literature review. According to published data by the Health Commision of Zhejiang Province, we collected the STRP assessment rsults of a total number of 12,036 residents. The inclusion cretria of these residents include: (1) 3rd-year residents. (2) taking STRP in the Zhejiang Province during 2016–2018 or 2017–2019. (3) the first time taking the clinical practice ability examination (CPAE) in 2018 or 2019. The results of 634 3rd-year residents from The Second Affiliated Hospital of Zhejiang University (SAHZU) were provided by the Department of Medical Education and were analyzed in depth. Three hundred and eight residents from SAHZU received normal training and took the CPAE in 2018, whereas 326 residets received PDCA and took the CPAE in 2019. PDCA is a program designed to improve the performance of residency in SAHZU. It includes the formulation and implementation of specific training plans, the check of effects, and continuous improvements. There was no change in the STRP assessment in these 2 years and the indicator of performance in the STRP assessment was the first pass rate (FPR). Statistical analyses were performed using Pearson's *chi-squared* test, Yates-corrected *chi-square* test, or Fisher's exact test (SPSS Statistics, version 25). A *P*-value of < 0.05 was considered significant.

**Results:** A total number of 6,180 and 5,856 examinees in the Zhejiang Province took the clinical practice ability examination in 2018 and 2019, respectively. In 2018, a total of 308 residents from 20 departments of the SAHZU took the STRP assessment. In 2019, a total of 326 residents from 22 departments of the SAHZU underwent the PDCA plan and took the STRP assessment. Compared with the results in 2018, the average FPR in the Zhejiang Province increased by 2.92% from 87.87 to 90.79% (*P* < 0.001). The FPR of the SAHZU increased by 7.88% from 85.06 to 92.94% (*P* = 0.001). In the SAHZU, the FPRs of the Department of Emergency and Department of Anesthesiology improved 34.51% (*P* = 0.024) and 20.36% (*P* = 0.004), respectively. There were no significant differences between the performances in the 2 years of the other 20 departments. There were improved results in the “Clinical Thinking and Decision-Making” and “Operation of Basic Skills” assessment stations with increases of 3.01% (*P* = 0.002) and 3.94% (*P* = 0.002), respectively. No statistically significant differences in the FPRs of the other six stations were found. The performances in all the stations in the final tests were better than in the stimulation tests (*P* < 0.001).

**Conclusions:** Although our sample size was relatively small, our results showed a small success of the PDCA plan in improving the quality of the STRP, especially for the residents in the Departments of Emergency and Anesthesiology. The PDCA plan also contributed to enhancing residents' abilities in the “Clinical Thinking and Decision-Making” and “Operation of Basic Skills” stations. Taken together, the PDCA plan may provide a practical framework for developing future training plans.

## Introduction

In the fight against disease, humans have established medicine and passed their long-term accumulated medical experience on to the next generation. The transfer of this knowledge and experience is typically through the continuous and unified process of medical education. After completing basic medical education, medical students are required to receive specialized training to deepen their knowledge and clinical skills in their specialization before becoming qualified junior doctors. Therefore, this training period, known as residency, plays an important role in medical education (1). Additionally, residents are the main force who undertake large amounts of basic medical work, such as collecting medical histories, recording the courses, and performing clinical operations under the guidance of superior doctors. Residents are first-line doctors who participate in the whole process of treating patients and thus should perform at high levels. Therefore, the standardized training of resident physicians (STRP) is designed to help improve the quality of residents (2, 3).

Some of the earliest countries to start STRP, including the United States (US) (3, 4), Germany (5, 6), and the United Kingdom (UK) (7, 8)), now have well-established and relatively complete STRP systems. Currently, different countries use different assessments for the STRP; however, the core competencies in the assessments are similar. In the US, medical students are required to pass the United States Medical Licensing Examination (USMLE)^1^ which is composed of Step 1, Step 2 Clinical Skills, Step 2 Clinical Knowledge, and Step 3 to get medical licensure. Step 1 assess the examinees' abilities to apply concepts from the basic sciences to medical practice. Step 2 Clinical Skills uses standardized patients (SP) to test examinees' ability to gather information from patients, perform physical examinations (PE), and communicate their findings to patients and colleagues. Step 2 Clinical Knowledge assesses the examinee's ability to apply medical knowledge, and understanding of clinical science essential for the provision of patient care under supervision, with an emphasis on health promotion and disease prevention. Step 3 assesses the examinee's ability to apply medical knowledge and understanding of biomedical and clinical science essential for the unsupervised practice of medicine, with emphasis on patient management in ambulatory settings. Analogous to the USMLE, there is a Professional and Linguistic Assessments Board (PLAB)^2^ test in the UK. The PLAB test^3^ focuses on professional knowledge and skills, patient safety, good partnerships with others and maintaining trust.

In China, the 3-year STRP has been continuously improved since 1993, and STRP has been vigorously carried out nationwide in recent years. Although the training standards vary among provinces and the training levels are different among hospitals, all residents are required to participate in provincial unified assessments in order to complete their training. Due to a lack of data from other provinces, this paper only describes the situation of STRP in the Zhejiang Province^4^ where the STRP assessment includes process assessment and graduation assessment. The former is a dynamic evaluation of the clinical competences and abilities of residents during the training, and the latter is a comprehensive evaluation of the overall effect of training. The graduation assessment is divided into two parts: the professional theory examination and the clinical practice ability examination (CPAE). The CPAE assesses the examinees' abilities to conduct standardized clinical operations and independently handle multiple common diseases in their major (Table 1). The first pass rate (FPR) of the CPAE is an indicator of the performance of the training program on the development of residents. Because of limited data, we only analyzed the results of 634 3rd-year residents from the SAHZU through studying the FPR of the CPAE in 2018 and 2019.

**Table 1:** Clinical practice ability examination.

**Station name**		**Assessment content**	**Assessment method**	**Numbers of examiners**	**Time**	**Total score**	**Pass**	**Comment**
Interpretation of clinical results		X-ray, CT, MRI, ultrasound, ECG, other laboratory tests	Man-machine dialogue exam	–	60 min	100	60	Automatic computer scoring
Patient consultation	Medical history collection	Medical history collection; doctor-patient communication skills	Clinical simulation	2	20 min	100	80	Selecting a disease recommended by the assessment regulations; gather information from patient and performing physical examinations; taking the average score of two points as the final score of this station, and any one of the scores below 80 considered to be unqualified.
	Physical examination	Special physical examination		2		100	80	
Medical document writing	First course record	1 First course record	Writing a record of the first course based on the patient's case at the examination station	2	15 min	100	80	Scoring based on assessment requirements
	Medical record	1 Medical record	1 Random medical record	2	–	100	80	
Clinical thinking and decision-making		Giving answers based on questions	Interviewing	2	20 min	100	80	Preparing corresponding questions and assessments according to the requirements of training standards and examination outlines for each subject
Operation of basic skills	CPR	CPR	Clinical simulation	2	10 min	100	80	Scoring based on assessment requirements
	Tracheal intubation	Tracheal intubation	Clinical simulation	2	10 min	100	80	
Operation of specialized skills		Performing operations based on cases	Clinical simulation	2	15 min	100	80	Preparing corresponding questions and assessments according to the training standards

## Methods

### The Compliance Statement

All research methods comprised in the study protocol were pursued/implemented in full compliance with pertinent guidelines, regulations, and applicable legislation in place.

### Ethical Approval

All participants provided written informed consent, and the study was conducted with the approval of the ethics committee of The Second Affiliated Hospital, School of Medicine, Zhejiang University.

### Inclusion Criteria

1. 3rd-year residents;
2. Taking STRP in SAHZU and other hospitals in the Zhejiang Province during 2016–2018 or 2017–2019;
3. The first time taking the CPAE in 2018 or 2019.

### Exclusion Criteria

1. Not 3rd-year residents;
2. Not taking STRP in the Zhejiang Province during 2016–2018 or 2017–2019;
3. Residents taking the make-up examination of the CPAE in 2018 or 2019.

### Information Collection

The information of all residents meeting the above inclusion and exclusion criteria was collected. Detailed information about the performance of residents from our hospital was provided by the relevant Departments and the FPR-associated data from the Zhejiang Province was collected online. There were 6,180 and 5,856 examinees that took the CPAE in 2018 and 2019, respectively. The number of examinees from our hospital was 308 and 326 in 2018 and 2019, respectively.

### The “Plan-Do-Check-Action” Plan

**Figure 1:**
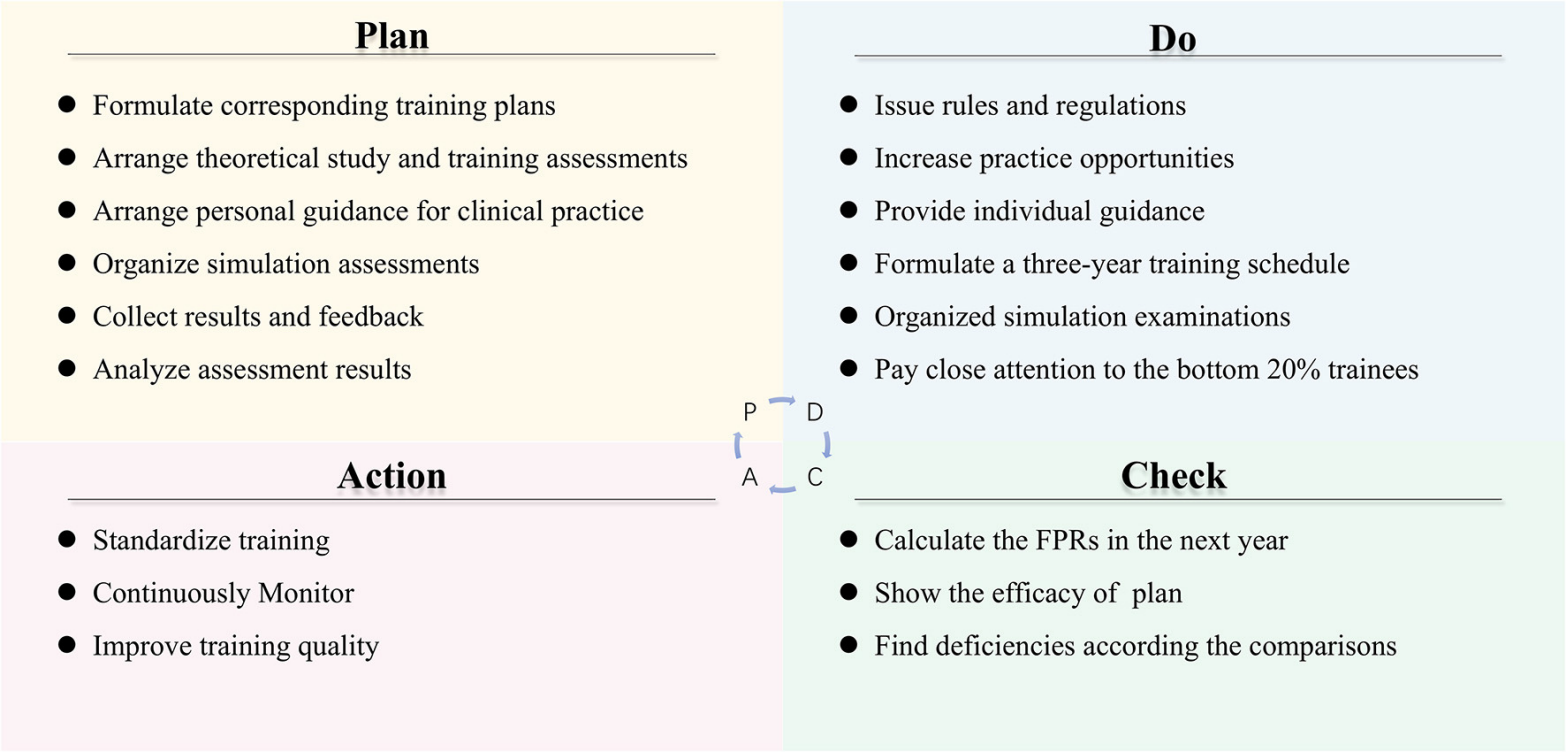
The PDCA plan.

The “Plan-Do-Check-Action” Plan (PDCA) (Figure 1) whose purpose was to comprehensively improve the quality of the STRP program and the FPR. PDCA included formulating plans, implementing the specific training plans, checking the effects by FPR and improving training quality.

#### Plan

A quality improvement team (QIT) composed of the Training Center and the Department of Medical Education (DME) was set up. The work of the QIT included: (1) formulate tailored training plans for residents in different departments; (2) arrange theoretical studies and training assessments; (3) arrange personal guidance for clinical practice in the training center; (4) organize simulation assessments before the final examination; (5) make trainees participate in the simulated examinations; (6) collect results and feedback; and (7) analyze the factors that contributed to the poor performance of trainees whose rank was in the bottom 20%. Subsequently, the QIT analyzed the results of examinees with an emphasis on the FPR before making further plans. There were constant adjustments in this step until a complete and practical solution was determined.

#### Do

Although time was always tight, the QIT tried to take several measures, such as increasing residents' attention, providing training venues and personal guidance, to improve the quality of residency training. In order to increase the attention of residents and supervisors on the STRP program, the DME issued rules and regulations for both residents and supervisors. For example, the assessment results of residents were factored into the supervisors' promotions, award applications, and annual evaluation by the department director. This measure helped to stimulate the enthusiasm of both the trainees and the supervisors. In addition, the Training Center provided more specialized training venues and extended training time to increase the number of practice opportunities for residents who were engaged in clinical work. Another measure was that residents who had problems in operating could ask for individual guidance from supervisors that were senior doctors from the corresponding department. For example, when residents were practicing tracheal intubation, anesthesiologists were arranged to provide individual guidance to residents to ensure that trainees have correctly mastered this skill. With the help of the Training Center and increased personal supervision, most trainees greatly improved. The fourth important measure was the formulation of a 3-year training schedule for 12 specialized skills (e.g., abdominal puncture, arteriovenous puncture, and defibrillation) training, which helped to strengthen clinical practice skills. Although this study focused on the performance of the 3rd-year residents (Appendix 1 showed a detailed timetable), all 1st-year residents were required to conduct the 12 specialized skills correctly. The assessments for the 3rd-year trainees emphasized “Medical History Collection,” “Physical Examination,” “Clinical Thinking,” and “Specialized Skills.”

In addition, the QIT organized a simulation residency examination to test all trainees' abilities during the last period (about 20 days) prior to the final examination. After obtaining the results of the simulation test, the QIT conducted a careful analysis of the trainees' performances and then retrained the bottom 20% of trainees with an emphasis on problematic stations. For stations like “Medical History Collection” and PE, the QIT used standardized patients (SP) (9), who were trained to consistently portray a wide variety of clinical cases, to provide a better understanding of the poor performances of the examinees. At the same time, the QIT invited excellent residents and SPs to take instructional videos on medical history collection and PE so that these referenced videos could provide standardized guidelines for those failed trainees. In all stations, examinees who failed in any station of the simulation examination received feedback from SP or supervisors before being re-evaluated. In addition, the QIT held a symposium for the bottom 20% of trainees so that they could figure out where their problems were arising from through face-to-face talks. Furthermore, the QIT informed the department to pay close attention to those trainees' performances. These steps enabled residents to have more opportunities to practice and resulted in significant progress.

#### Check and Action

In this step, the QIT calculated the FPR in the next year to find the efficacy of the PDCA plan and find deficiencies according to the comparisons with the results of previous years. The last step was “Action,” which primarily included training standardization, continuous monitoring, and quality improvements. In this stage, it was proposed to standardize the 3-year training by formulating the “Layered Training Program for Clinical Practice Competence of Residents” according to the national requirements and standards as well as to conduct more specific training outlines in the different departments. Detailed clinical practice ability grading tables (Appendix 2) were provided. It was also suggested to formulate the “Master Plan for the Training of Residents' Skills” to annually test the trainees' clinical abilities. Continuous monitoring was associated with extending the time of the PDCA plan and continued data collection. Some deficiencies were taken into consideration for quality improvements. First, residents were engaged in clinical work and they probably missed some of the training time. In regards to time constraints, the QIT extended the practice training time. Second, there was an increasing number of trainees, which brought difficult to supervision. To solve this problem, the QIT created a useful information system to make comprehensive records to track the training process of trainees and implemented a staggered, centralized training to reduce the burden on the trainers.

### Statistical Analysis

Statistical analyses were performed using Pearson's *chi-squared* test, Yates-corrected *chi-square* test, or Fisher's exact test (IBM SPSS Statistics, version 25). A *P*-value level of < 0.05 was considered significant.

## Results

### The First Pass Rate in 2018

**Figure 2:**
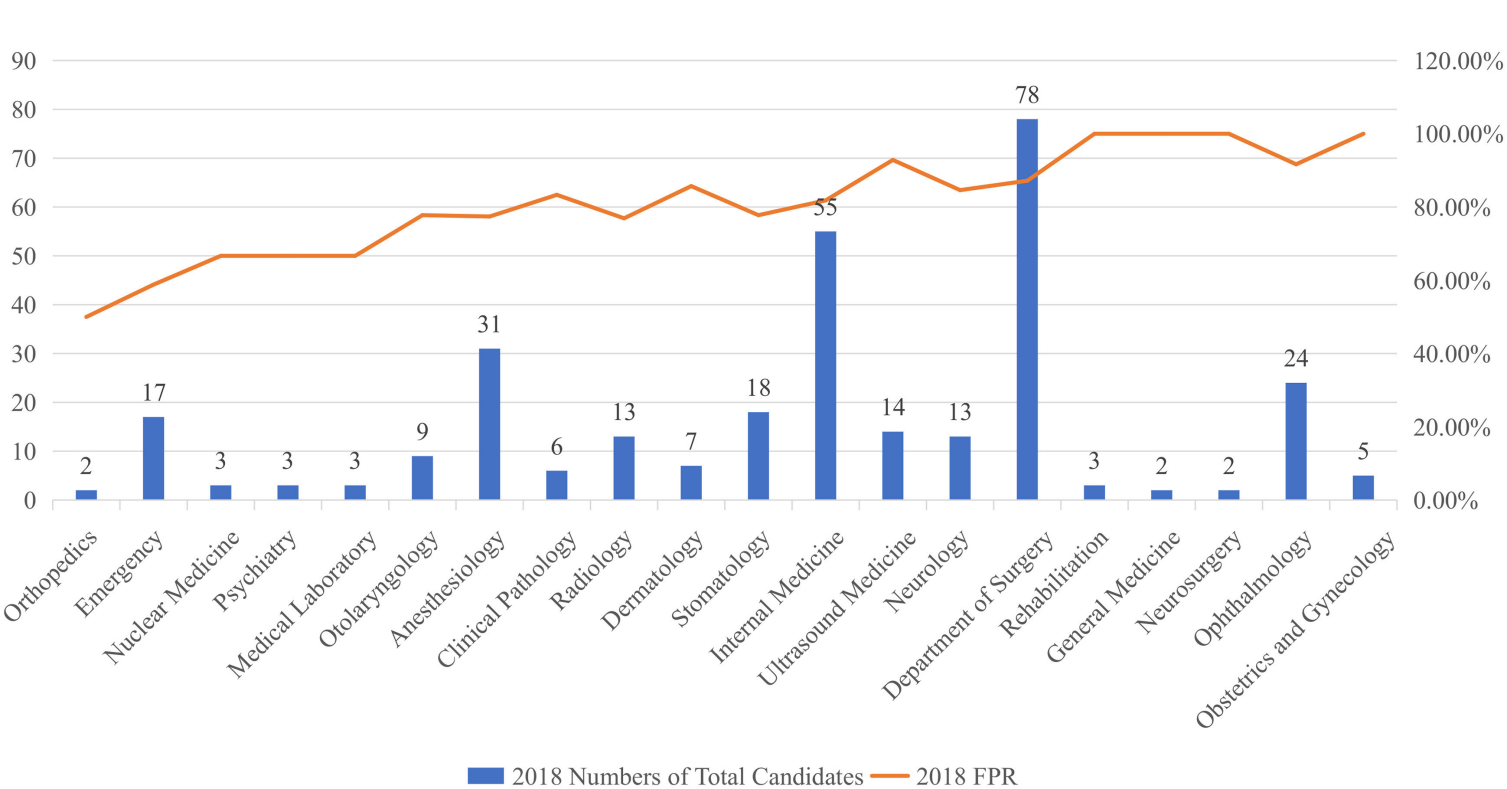
Results of first pass rate in different departments in 2018.

In 2018^5^, 5,430 examinees out of a total of 6,180 examinees that took the CPAE passed, which resulted in an FPR of 87.87%. Of the 6,180 total examinees, 308 of them were from our hospital, and 262 examinees passed. The FPR of examinees from our hospital was 85.06%, which was lower than the average FPR in the Zhejiang Province. When the performance on the CPAE was examined at the station level, the two highest FPRs from our hospital were in “Medical Records” and “Medical History Collection.” Both FPRs were higher than the average FPR in the Zhejiang Province. However, the FPRs of the other four stations, including “Operation of Specialized Skills,” “Medical Document Writing,” “Clinical Thinking and Decision-Making,” and “Operation of Basic Skills,” were below the corresponding average FPRs in the Zhejiang Province (Table 2). Examinees from our hospital did the worst in the “Operation of Basic Skills” station out of the eight stations, with the lowest FPR of 95.45%. Additionally, FPRs for the complete CPAE varied among departments. The Department of Rehabilitation, Department of General Medicine, Department of Neurosurgery, and Department of Obstetrics and Gynecology all performed well with FPRs of 100%; however, the Department of Orthopedics performed the worst with an FPR of 50%. In addition, the Department of Ultrasound Medicine and Department of Ophthalmology had FPRs of over 90% (Figure 2).

**Table 2:** Results of first pass rate in different stations in 2018.

**Station name**	**Numbers of total examinees**	**Numbers of qualified examinees**	**FPR**	**Average FPR in Zhejiang Province**
Medical record	302	0	100.00%	99.98%
Medical history collection	302	3	99.01%	98.93%
Operation of specialized skills	302	3	99.01%	98.99%
Physical examination	302	7	97.68%	97.91%
Medical document writing	254	6	97.64%	98.15%
Clinical thinking and decision-making	299	9	96.99%	97.45%
Interpretation of clinical results	308	12	96.10%	96.99%
Operation of basic skills	308	14	95.45%	97.85%

### The First Pass Rate in 2019 and Improvements

**Figure 3:**
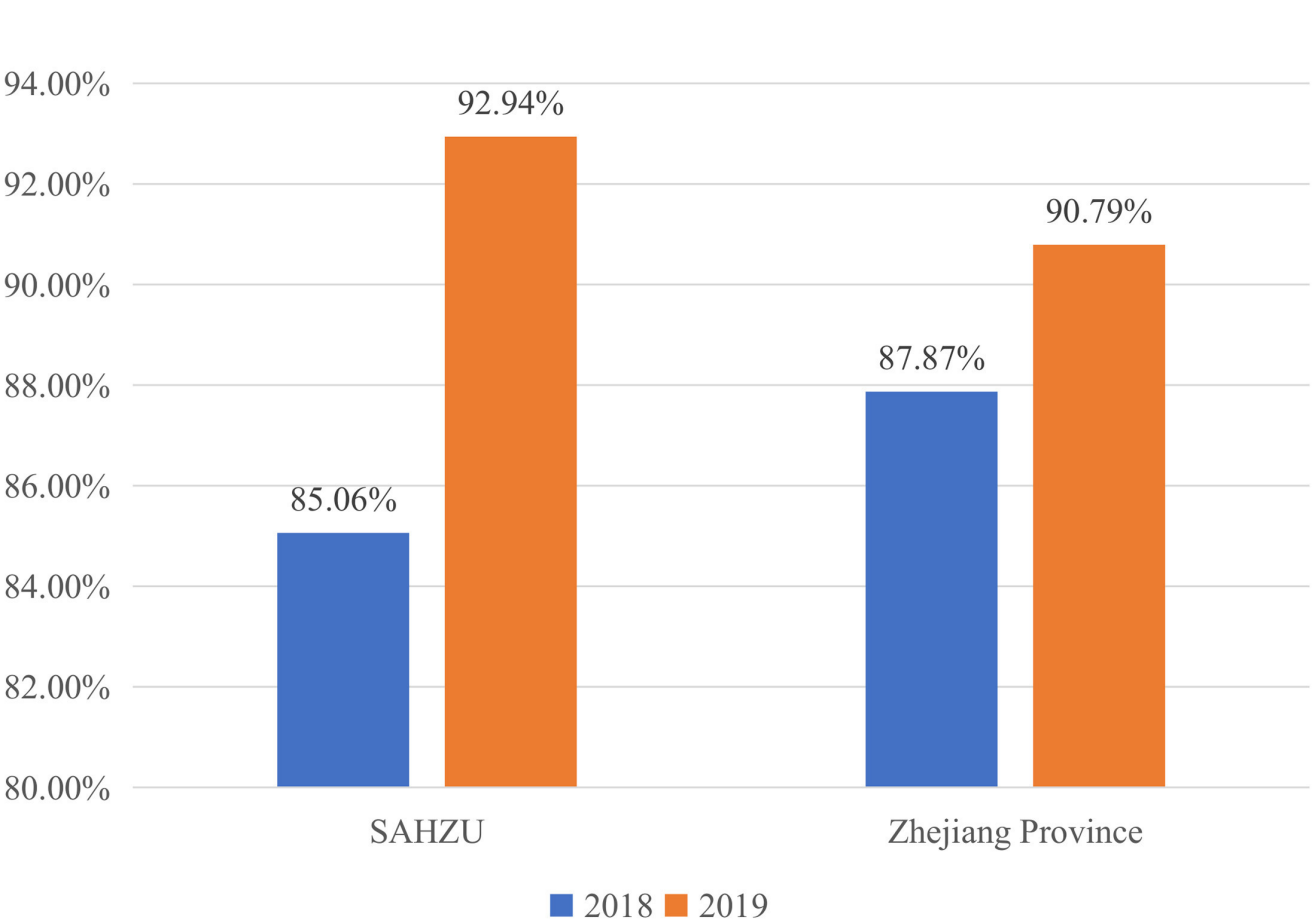
The first pass rate in 2018 and 2019.

**Figure 4:**
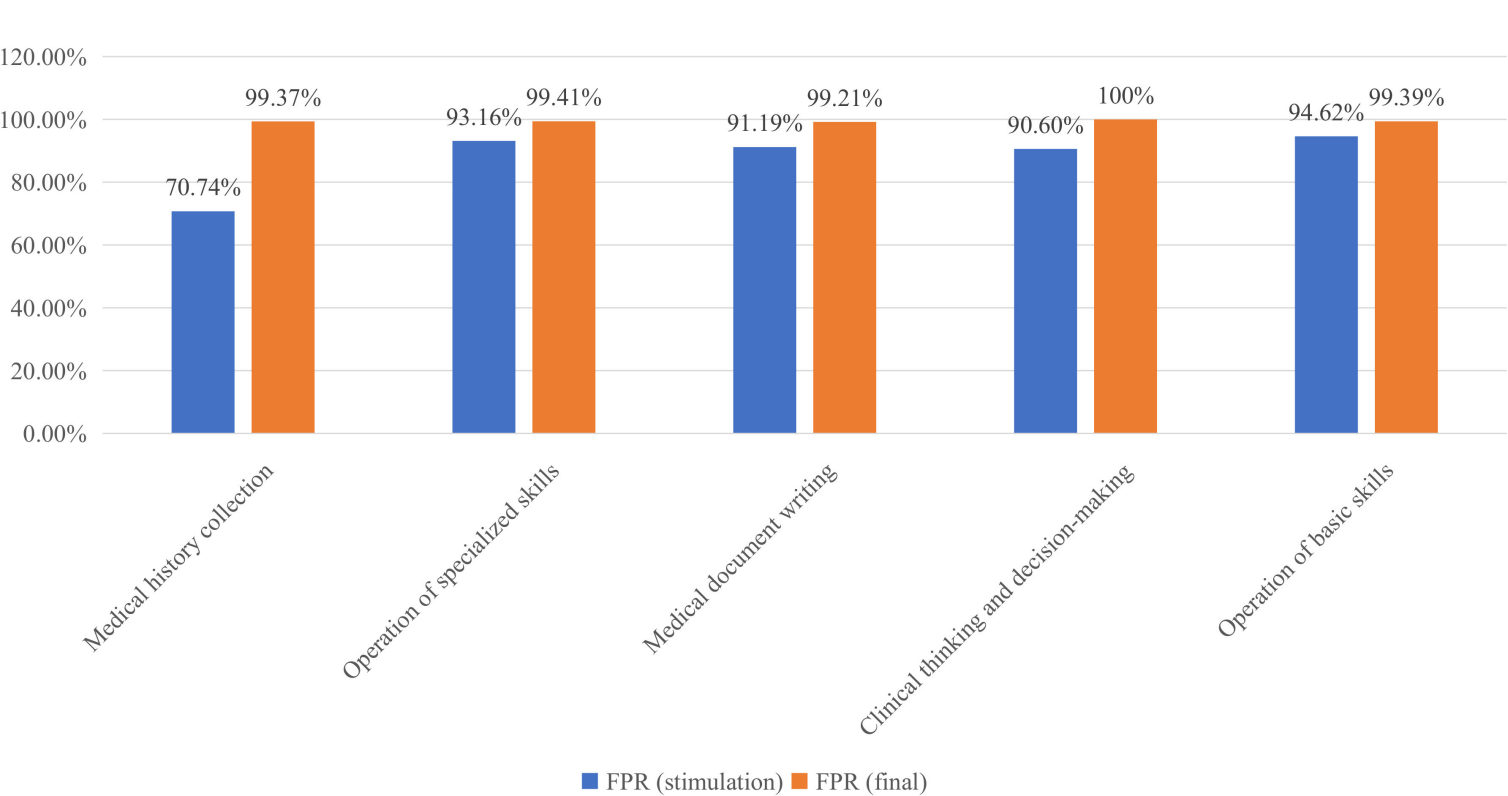
Results of stations in stimulation and final examinations in 2019.

In 2019, 5,317 examinees out of a total of 5,856^6^ examinees that took the CPAE in the Zhejiang Province passed. There were a total of 326 examinees from our hospital. Compared with the results in 2018, the average FPR in the Zhejiang Province increased by 2.92% from 87.87 to 90.79% (*P* < 0.001). The FPR of examinees from the SAHZU increased by 7.88% from 85.06% in 2018 to 92.94% in 2019 (*P* = 0.001) (Figure 3). In the SAHZU, the FPRs of the Department of Emergency and Department of Anesthesiology improved 34.51% (*P* = 0.024) and 20.36% (*P* = 0.004), respectively. There were no other significant differences between the FPRs in 2018 and 2019 in the other 20 Departments (Table 3). When the performance on the CPAE was broken down by assessment stations, the FPRs for “Clinical Thinking and Decision-Making” and “Operation of Basic Skills” increased 3.01% (*P* = 0.002) and 3.94% (*P* = 0.002), respectively, from 2018 to 2019. No significant differences in the FPRs for the other six stations were found (Table 4). In the process of “Plan-Do-Check-Action” (PDCA) plan, it was observed that the FPR of the final examination in all stations was higher than that in the stimulation examination (*P* < 0.001), particularly in the “Medical History Collection” assessment where the FPR improved 28.63% (Figure 4).

**Table 3:** The first pass rate associated with Departments in 2018 and 2019.

**Department**	**FPR in 2018 *n* (%)**	**FPR in 2019 *n* (%)**	***χ^2^***	***P*-Value**
Pediatrics	/	1 (100)	/	/
Radiation oncology	/	1 (100)	/	/
Orthopedics	2 (50)	7 (85.71)	0.417	0.284
Emergency	17 (58.82)	15 (93.33)	0.041	0.024
Nuclear medicine	3 (66.67)	2 (100)	1	0.361
Psychiatry	3 (66.67)	2 (100)	1	0.361
Medical laboratory	3 (66.67)	2 (100)	1	0.361
Otolaryngology	9 (77.78)	8 (100)	0.471	0.156
Anesthesiology	31 (77.42)	45 (97.78)	6.061	0.004
Clinical pathology	6 (83.33)	11 (100)	0.353	0.163
Radiology	13 (76.92)	12 (91.67)	0.593	0.315
Dermatology	7 (85.71)	8 (100)	0.467	0.268
Stomatology	18 (77.78)	32 (90.63)	0.692	0.209
Internal medicine	55 (81.82)	56 (92.86)	2.148	0.08
Ultrasound medicine	14 (92.86)	15 (100)	0.483	0.292
Neurology	13 (84.62)	11 (90.91)	1	0.642
Department of surgery	78 (87.18)	55 (87.27)	0	0.987
Rehabilitation	3 (100)	4 (100)	/	/
General medicine	2 (100)	3 (100)	/	/
Neurosurgery	2 (100)	4 (100)	/	/
Ophthalmology	24 (91.67)	23 (82.61)	0.416	0.352
Obstetrics and gynecology	5 (100)	9 (88.89)	1	0.439

**Table 4:** The first pass rate associated with stations in 2018 and 2019.

**Station name**	**FPR in 2018 *n* (%)**	**FPR in 2019 *n* (%)**	***χ^2^***	***P*-Value**
Medical record	302 (100)	315 (100)	/	/
Medical history collection	302 (99.01)	315 (99.68)	0.299	0.294
Operation of specialized skills	302 (99.01)	315 (99.68)	0.299	0.294
Physical examination	302 (97.68)	315 (99.37)	1.98	0.081
Medical document writing	254 (97.64)	315 (99.21)	1.143	0.154
Clinical thinking and decision-making	299 (96.99)	254 (100)	0.001	0.002
Interpretation of clinical results	308 (96.10)	313 (95.09)	0.384	0.535
Operation of basic skills	308 (95.45)	326 (99.39)	8.419	0.002

## Discussion

To better understand the current implementation of STRP and find the reasons for the unsatisfactory performance, DME took a series of measures, such as organizing meetings for in-depth discussion and conducting regular face-to-face conversations with resident representatives from all departments in our study. The primary factors were vague training plans, time constraints, lack of teaching resources, and lack of practice opportunities.

### Vague Training Plan

Because our hospital is a large comprehensive teaching hospital, there is a lot of teaching and training work that is needed for residents. However, STRP was not complete in China at the time and there were no specific training plans for residents from different departments in our hospital. In fact, the training of medical personnel, such as interns, residents, and fellows, in the hospitals of China was vague and untargeted. During the STRP process, the residents did not know the clear requirements for themselves. When they were working and getting trained in different departments, they have no clear goals or plannings. This may have resulted in some residents not having significant improvements after 3 years. Therefore, a clearer training plan for personnel in different disciplines or types was required.

### Time Constraints and Burnout

In medical school, students are focused on studying. However, during the residency stage, residents not only need to complete countless amounts of paperwork and practice professional skills to improve their ability, but they also need to complete many other tasks (e.g., participating in scientific research or academic forums) to keep them up-to-date on the latest developments. In addition, heavy clinical burdens and tough academic studying make it difficult for residents to balance time and energy to perform well in all aspects, which drives them to pay the most attention to completing work rather than extending clinical knowledge or improving clinical skills. Thus, all of these demands lead to burnout being a common problem for residents, especially those in surgical residency training programs. Hu et al. did a cross-sectional national survey of 7,409 residents and found that 38.5% of these residents reported burnout symptoms weekly (10). In addition, Lichstein et al. conducted an orthopedic educational survey study that showed that 52% (342 of 661) of residents reported burnout and that the factors causing burnout included unmanageable work volume (OR 3.13; 95% CI, 1.45–6.67; *P* < 0.01) and lack of exercise (OR 1.69; 95% CI, 1.08-2.70; *P* = 0.02) (11). In our hospital, there was a large volume of work as there were 92,553 inpatient operations and 186,102 discharges in 2019. In addition, the average hospital stay was 6.09 days. Even though there was no detailed data about the wellness of

the residents in our hospital, most residents had complained about time constraints. Furthermore, time spent on clinical improvement was greatly compressed and residents' operation time was limited, which may have made it hard for residents to meet the training requirements. Therefore, residents should have good time management plans and the departments should have reasonable divisions of labor.

### Lack of Teaching Resources and Practice Opportunities

There were many factors that resulted in the deficiency of teaching resources. First, the rapid growth in the number of residents who were required to have STRP and the lack of available teaching resources created a demand that was higher than supply. Second, senior doctors devoted themselves to heavy clinical workloads and they might not have had extra time to focus on clinical teaching, which may have resulted in them ignoring residents. Consequently, the trainees were inactive in raising or analyzing problems resulting in making their clinical thinking and emergency response ability rather weak. Third, due to the uneven levels of teachers' professional skills and clinical teaching skills, it was hard to achieve homogeneity in the training process. Fourth, although the training program was characterized by “learning in practice,” the obtained training in practice was not satisfactory due to the lack of guidance from experienced doctors. Fifth, many of the departments had adopted the strategy of “one size fits all” in the arrangement of training time in order to save time and manpower, which led to poor quality of training. Under the combined influence of these factors, residents became indifferent to department rotations and gradually lost initiative and enthusiasm toward the STRP program. Herein, a reasonable allocation of teacher resources and an increase of examination practice time in daily work were worth discussing.

Yet, there are no complete and specific training plans for the different departments and this deserves our continued efforts. In addition, the lack of wellness among residents is troubling. Therefore, educational leaders of the STPR program should pay attention to residents' burnout and mental health as well as provide access for health maintenance in order to foster a positive and comfortable environment for trainees.

Despite factors such as time constraints and heavy clinical workloads, the PDCA plan still needs to be improved as follows: (1) develop separate training plans for each department; (2) provide better models for training; (3) cooperate with clinical departments to improve trainees' clinical abilities rather than just work hard to pass the STRP examination; (4) pay attention to trainees' physical and mental health because they are too busy; (5) reward departments for excellent performance to keep their enthusiasm in teaching and training. Taken together, we propose that the PDCA plan will become more and more useful in the future and it may contribute to the development of medical education in China or other countries.

## Conclusion

STRP has become an important part of medical education and the FPR is considered as an indicator that shows the effect of STRP implementation in Chinese hospitals. With an increasing number of medical students and trainees, how to improve the FPR and cultivate outstanding clinicians using limited teaching resources is a question that requires constant thinking. Here, we used a practical approach to this problem, which is the PDCA plan. Although the sample size was small and the observation time was short, we witnessed success of the PDCA plan through an increased FPR (Table 3). Additionally, the simulation examination of the PDCA plan helped to figure out the reasons for poor performance. As residents were busy working and many of them may miss the stimulation emanation, it was difficult to make this examination the same as the final one. As the “Medical Records” and “Interpretation of Clinical Results” were evaluated on their performance in daily work, assessments of five stations (“Medical History Collection,” “Operation of Specialized Skills,” “Medical Document Writing,” “Clinical Thinking and Decision-Making,” and “Operation of Basic Skills”) were organized. In fact, the number of participants in the stimulation examination was less than the number in the final examination because residents were too busy to attend it. However, based on collected information from stimulation examination we were able to identify problems and poor performances in some stations, including “Medical History Collection” and “Clinical Thinking and Decision-Making” (Figure 4). After taking several measures (e.g., inviting SPs, watching videos, and holding seminars), trainees performed better on the final examination (Figure 4).

## Limitation

The most conspicuous limitation is the small sample size. Due to data confidentiality and the lack of open database for SRTP examination in China, we did not have access to the detailed results of residents' performances at other hospitals. Therefore, we collected data from our own center through the simulation and actual SRTP examinations, which resulted in small sample size. In future, we hope that teaching hospitals can cooperate with others and strengthen data sharing to achieve better medical training performances. In addition, there were different numbers of residents, ranging from 1 to 78, in different departments because of the Chinese department setting system and the strength of the hospital. This brings statistical errors and makes the results unconvincing. However, an evaluation of these departments (e.g., Pediatrics, Nuclear Medicine, and Psychiatry) was necessary because there were a number of residents in those departments. In the future, multicenter and provincial or national studies involving a greater number of examinees and detailed data are required to better understand the situation of the STPR program across China. The second limitation is the data collected from the simulation examination. The missing data on “Medical Records” and “Interpretation of Clinical Results” may influence identifying the efficacy of the PDCA plan. Furthermore, due to busy and uncontrollable work schedules, the number of participants in the simulation examination was less than that in the final one. In the following year, we will find a better time to schedule the simulation so that more residents can participate. The third limitation of this study is the short observation time. In this study, we observed results from 2 years and saw a slight increase of FPR. However, in some exceptional circumstances (i.e., the pandemic of COVID-19), cultivating high-quality juniors in a short time is important for the society. A slight improvement in STRP may be of great significance to regions or countries. In fact, we will conduct the PDCA plan in the following years with an enrollment of the 1st-, 2nd- and 3rd-year residents, and collect more comprehensive data. We will continue to develop STRP and improve it to make the STRP more objective and reliable. Last but not least, we hope that our educational experience can bring benefits to other hospitals though STRP is still under exploration.

## Data Availability Statement

The raw data supporting the conclusions of this article will be made available by the authors, without undue reservation.

## Ethics Statement

The studies involving human participants were reviewed and approved by The Second Affiliated Hospital, School of Medicine, Zhejiang University. The patients/participants provided their written informed consent to participate in this study.

## Author Contributions

BT and AS conceptualized the research project. DL wrote the paper and made the original figure and tables. AS, MY, FZ, and BT critically revised the texts and figures. AS and BT supervised the research and led the discussion. All authors read and approved the final manuscript.

## Conflict of Interest

The authors declare that the research was conducted in the absence of any commercial or financial relationships that could be construed as a potential conflict of interest.

## Footnotes

^1^https://www.usmle.org/pdfs/bulletin/2020bulletin.pdf

^2^https://www.gmc-uk.org/registration-and-licensing/join-the-register/plab/a-guide-to-the-plab-test

^3^https://www.gmc-uk.org/-/media/documents/good-medical-practice—english-20200128_pdf-51527435.pdf?la=en&hash=DA1263358CCA88F298785FE2BD7610EB4EE9A530

^4^http://zjyxjy.zjwjw.gov.cn/Home/Content?news_id=ED09CC5C-AF20-40EE-8905-AB8A00F38A3D

^5^http://www.med66.com/zhuyuanyishi/zhejiang/cd1809097926.shtml

^6^http://www.zjyxjl.org.cn/detail.aspx?newsId=1354

## Supplementary Material

Appendix 1 Arrangements of 12 operation trainings.

Appendix 2 Layered training program for clinical practice competence of residents.

## Abbreviations

CPAE: clinical practice ability examination
DME: the Department of Medical Education
FPR: first pass rate
PDCA: Plan-Do-Check-Action
PLAB: Professional and Linguistic Assessments Board
QIT: quality improvement team
SAHZU: The Second Affiliated Hospital of Zhejiang University
SP: standardized patient
STRP: the standardized training of resident physicians
USMLE: the United States Medical Licensing Examination.
