# Supplementary material for: The “Plan-Do-Check-Action” Plan Helps Improve the Quality of the “Standardized Training of Resident Physicians”: An Analysis of the Results of the First Pass Rate
Source: Front Public Health. 2021 Feb 23;8:598774. doi: 10.3389/fpubh.2020.598774 (PMC7940191; doi:10.3389/fpubh.2020.598774)
Supplement: Appendix 1 — Arrangements of 12 operation trainings. [file Data_Sheet_1.pdf]

The Second Stage Skills Operation Training Schedule of Resident Physicians in 2019

Operational items	Week	Defibrillation	Abdominal puncture	Bone marrow biopsy	Spine injury handling	Arteriovenous puncture	Abscess incision	Lumbar puncture	Debridement	Put on and take off the isolation gown	Cut and knot	Three-cavity two-cyst tube placement	Thoracic puncture
		Cardiology	Endocrine/ Nephrology	Hematology	Orthopedics	Anesthesia	Oncology	Neurology	Urology	Neurosurgery	Surgery	Gastroenterology	Respiratory Medicine
Training classroom		505	506	508	508	505	506	505	506	503	506	510	505
2019.2.26	Tuesday	**Group 1**	**Group 2**	**Group 3**									
2019.2.27	Wednesday				Group 4	Group 5	Group 6						
2019.2.28	Thursday							Group 7	Group 8	Group 9			
2019.3.01	Friday										Group 10	Group 11	Group 12
2019.3.5	Tuesday	Group 10	Group 11	Group 12									
2019.3.6	Wednesday				**Group 1**	**Group 2**	**Group 3**						
2019.3.7	Thursday							Group 4	Group 5	Group 6			
2019.3.8	Friday										Group 7	Group 8	Group 9
2019.3.12	Tuesday	Group 7	Group 8	Group 9									
2019.3.13	Wednesday				Group 10	Group 11	Group 12						
2019.3.14	Thursday							**Group 1**	**Group 2**	**Group 3**			
2019.3.15	Friday										Group 4	Group 5	Group 6
2019.3.19	Tuesday	Group 4	Group 5	Group 6									
2019.3.20	Wednesday				Group 7	Group 8	Group 9						
2019.3.21	Thursday							Group 10	Group 11	Group 12			
2019.3.22	Friday										**Group 1**	**Group 2**	**Group 3**
2019.3.26	Tuesday	**Group 1**	**Group 2**	**Group 3**									
2019.3.27	Wednesday				Group 4	Group 5	Group 6						
2019.3.28	Thursday							Group 7	Group 8	Group 9			
2019.3.29	Friday										Group 10	Group 11	Group 12
2019.4.02	Tuesday	Group 10	Group 11	Group 12									

2019.4.03	Wednesday				**Group 1**	**Group 2**	**Group 3**						
2019.4.04	Thursday							Group 4	Group 5	Group 6			
2019.4.09	Tuesday										Group 7	Group 8	Group 9
2019.4.10	Wednesday	Group 7	Group 8	Group 9									
2019.4.11	Thursday				Group 10	Group 11	Group 12						
2019.4.12	Friday							**Group 1**	**Group 2**	**Group 3**			
2019.4.16	Tuesday										Group 4	Group 5	Group 6
2019.4.17	Wednesday	Group 4	Group 5	Group 6									
2019.4.18	Thursday				Group 7	Group 8	Group 9						
2019.4.19	Friday							Group 10	Group 11	Group 12			
2019.4.23	Tuesday										**Group 1**	**Group 2**	**Group 3**
2019.4.24	Wednesday	**Group 1**	**Group 2**	**Group 3**									
2019.4.25	Thursday				Group 4	Group 5	Group 6						
2019.4.26	Friday							Group 7	Group 8	Group 9			
2019.4.30	Tuesday										Group 10	Group 11	Group 12
2019.5.07	Tuesday	Group 10	Group 11	Group 12									
2019.5.08	Wednesday				**Group 1**	**Group 2**	**Group 3**						
2019.5.09	Thursday							Group 4	Group 5	Group 6			
2019.5.10	Friday										Group 7	Group 8	Group 9
2019.5.14	Tuesday	Group 7	Group 8	Group 9									
2019.5.15	Wednesday				Group 10	Group 11	Group 12						
2019.5.16	Thursday							**Group 1**	**Group 2**	**Group 3**			
2019.5.17	Friday										Group 4	Group 5	Group 6
2019.5.28	Tuesday	Group 4	Group 5	Group 6									
2019.5.29	Wednesday				Group 7	Group 8	Group 9						
2019.5.30	Thursday							Group 10	Group 11	Group 12			
2019.5.31	Friday										**Group 1**	**Group 2**	**Group 3**
