# Supplementary material for: The “Plan-Do-Check-Action” Plan Helps Improve the Quality of the “Standardized Training of Resident Physicians”: An Analysis of the Results of the First Pass Rate
Source: Front Public Health. 2021 Feb 23;8:598774. doi: 10.3389/fpubh.2020.598774 (PMC7940191; doi:10.3389/fpubh.2020.598774)
Supplement: Appendix 2 — Layered training program for clinical practice competence of residents. [file Data_Sheet_2.pdf]

Clinical Practice Ability

Scoring Tables for Each Examination Station

Standardized Training of Resident Physicians in Zhejiang Province

(Internal medicine and surgery)

Clinical Practice Ability Scoring Table

Scoring Table for Medical History Collection

Candidate name		Candidate Number	
Training discipline		Training base	
Assessment base		Assessment time	
**Scoring items**	**Criteria**	**Standard score**	**Score**
General Condition	Introduce yourself (1 point for each item: name, position or responsibility).	2	
	Ask about the patient's basic information such as name, age, medical record number, occupation, birth place (1 point for each item).	5	
History of present illness	Chief compliant: Onset and time (2.5 points for each item)	5	
	Causes of onset	2	
	Main symptoms and the course of disease (4 points each)	8	
	Accompanying symptoms and negative identification symptoms (2.5 points each)	5	
	Diagnosis and treatment (diagnosis and treatment unit, examination results, treatment measures, medication conditions and treatment effects, etc., 1 point per item)	5	
	Current general condition (diet, sleep, urine, weight change, etc., 1 point for each item)	5	
Past medical history	Previous illness and long-term medication history, surgical trauma history, food and drug allergy history, infectious disease history, blood transfusion history, vaccination history, etc. (2 points per item)	12	
Personal history	Smoking history, drinking history, marriage and childbirth history (women's history of menstrual marriage and childbirth), etc. (2 points per item)	6	
Family history	Similar illnesses in the family, two-line and three-generation genetic disease history (2.5 points each)	5	
Skills of Inquiry	Inquiry is organized and focused (4 points), and there is a differential diagnosis thinking (4 points).	8	
	The language of inquiry is appropriate, without over-guidance or blame.	5	
	Show sympathy and encouragement to the patient.	5	
Doctor-patient communication	Kind doctor-patient communication attitude and professional expression	5	
	Summarize medical history information and take the initiative to communicate with patients or family members (5 points for each item).	10	
	Respond to patient questions and provide professional advice patiently.	5	
	Understand the patient's family's financial ability to pay and medical insurance.	2	
Total		100	

Examiner's signature:

Clinical Practice Ability Scoring Table

Scoring Table for Physical Examination

Candidate name		Candidate Number		
Training discipline		Training base		
Assessment base		Assessment time		
**Scoring items**	**Criteria**	**Standard score**	**Score**	

Scoring items	Criteria	Standard score	Score
General Examination	**General situation and head and neck (20 points. Incorrect operation, 1-point deduction each time)**		
	Warm hands and stethoscope. Prepare various examination tools (1 point), and inform the patient of intention (1 point).	2	
	Eyes: conjunctiva (observation of hyperemia, 1 point), sclera (observation of yellow staining, 1 point), pupil reflection of light (2 points)	4	
	Superficial lymph nodes of the head and neck: submandibular, subchin, neck, supraclavicular lymph nodes (1 point each)	4	
	Oral mucosa, tongue, pharynx, tonsils (1 point each)	4	
	Face and skin: skin with or without yellow staining, cyanosis, rash, paleness (0.5 points each)	2	
	Neck: tracheal position, neck stiffness (can be checked simultaneously with lymph nodes, 2 points each)	4	
	**Chest (20 points. Incorrect action, 1-point deduction each time)**		
	(Inspection and palpation) Chest wall skin (1 point), examination of tenderness (1 point), respiratory symmetry (2 points)	4	
	(Percussion) Comparison of upper, lower, left, and right lung (no percussion of lung apex and lower lung activity)	4	
	(Auscultation) At least 2 breathing cycles (including chest, lateral chest, and back auscultation. Check each area and make comparisons (up and down, left and right). 2 points for comparison	3	
	(Inspection and palpation) Apical pulse (2 points for tangential inspection)	4	
	(Auscultation) Auscultation of 5 valve auscultation areas in sequence (1 point for each area)	5	
	**Abdomen (20 points. 2 points for correct abdominal examination sequence; 1-point deduction for each incorrect operation)**		
	(Inspection) Abdominal morphology	2	
	(Auscultation) Bowel sounds (2 points per item for correct auscultation site and the dictation duration)	4	
	(Percussion) Routine percussion in a counter clockwise order (no splenic percussion required)	4	
	(Palpation) Shallow palpation and deep palpation of the abdomen (pay attention to the tenderness of the patient, 2.5 points each), liver and spleen palpation (2.5 points each)	10	
	**Nervous system and limbs (20 points. Incorrect movements, 1-point deduction each time)**		
	Tendon reflex, Brudzinski sign, Kernig sign, Babinski sign (2 points each)	8	
	Muscle strength of limbs and bilateral knee reflexes (2 points each)	4	
	Compression examination of lower extremity oedema (check both ankles and anterior tibia, 2 points each)	4	
	Bilateral contrast examination of dorsal artery and radial artery pulse (2 points for each item)	4	
Specific examinatio	Combine the general physical examination and specific examination according to the medical history (5 points).	15	

Clinical Practice Ability Scoring Table

n	Choose the correct physical examinations and complete them in a sufficient and reasonable way (5 points).		
	Specific physical examination steps can reflect diagnosis ideas and identify key points (5 points).		
Overall evaluation	Standardized physical examination (1 point), proficient and ordered examination (1 point), appropriate humane care (2 points), and timely completion (1 point)	5	
Total		100	

Examiner's signature:

Clinical Practice Ability Scoring Table

Scoring Table for the First Course Record

Candidate name		Candidate Number		
Training discipline		Training base		
Assessment base		Assessment time		

**Scoring items**	**Criteria**	**Standard score**	**Score**
Subjective brief medical history (S)	A brief summarization of the history of present illness with highlights	10	
	Consistent with medical history information	5	
Objective medical examination records/ results (O)	Correct descriptions of physical examinations meaningful for disease diagnosis	5	
	Correct descriptions of the auxiliary examination results associated wiht the disease	5	
Diagnostic evaluation and differential diagnosis (A)	The diagnosis basis is brief and complete.	5	
	The diagnosis basis is not stacked, and the content is not empty.	5	
	Each diagnosis has a medical history, physical examination, and auxiliary examination basis.	5	
	Accurate diagnosis with reasonable primary and secondary orders.	10	
	The differential diagnosis is combined with the main diagnosis of the patient, and the analysis is organized.	10	
	No differential diagnosis unrelated to diagnosis	5	
Treatment plan (P)	The diagnosis and treatment plan are in line with the basic treatment principles.	10	
	The treatment plan corresponds to the relevant diagnosis.	10	
	The diagnosis and treatment plan reflects the individualized principle of each patient's condition.	5	
Overall	The overall writing of the first course of history is concise, and the clinical thinking is clear.	5	
	Neat handwriting	5	
Total score		100	

Examiner's signature:

Clinical Practice Ability Scoring Table

Scoring Table for the Major Medical Record

Candidate name		Candidate Number	
Training discipline		Training base	
Assessment base		Assessment time	

Scoring items		Criteria	Standard score	Score
Admission record	General information	Name, gender, age, occupation, etc.	2	
	Chief complaint	Concise and complete chief complaint	2	
	History of present illness	Time of onset, predisposing factors, symptoms, mitigating factors, treatment, negative symptoms for differential diagnosis, and general condition after the illness onset.<br>A record of other diseases which are not related to illnesses this time and require further treatment.	8	
	Past medical history	Past medical history, personal history, allergy history, marriage and childbirth history, menstrual history, family history, etc.	3	
	Physical examination	Complete general physical examination records of all major systems and accurate physical examination records of specialists and negative signs for disease identification	4	
	Auxiliary examination	The main auxiliary examinations are related to the disease and their results.	2	
	Diagnosis	The preliminary diagnosis is accurate.<br>The correction and supplementary records are recorded in the course record accordingly.	4	
First course record	Medical records	The summary is brief and clear.	4	
	Diagnose based on	Each diagnosis is supported by medical history, physical examination, and auxiliary examination.	6	
	Differential diagnosis	The analysis is organized and logical.	4	
	Treatment plan	Propose specific inspection and treatment arrangements.	6	
Course record	Time	Critical illness > 1 time / day, serious illness> 1 time / 2 days, stable condition 1 time / 3 days	5	
	Content	Accurately show the changes of patient's condition and the process of diagnosis and treatment.<br>Record and analyze the results of the auxiliary results.<br>Record important changes of treatments (antibiotic and special medicine).<br>Complete handover shift records, transfer records, and stage summary on time.<br>Complete records of necessary operations and rescue records on time.<br>Record detailed case discussion records in a clear and focused way (5 points per item).	30	
	Ward round record of superior doctor	Complete within the required time (twice a week for attending physician and once a week for chief).<br>Clear and useful records with focuses	5	
Discharge	General	Name, gender, age, date of admission, date of discharge,	2	

Clinical Practice Ability Scoring Table

record	information	hospital stay		
	Admissions	Concise and clear record, with a strong focus, and a reasonable diagnosis at admission	2	
	Treatment	The changes in the patient's condition during the hospitalization, the examination results, the treatment processes are clearly stated.	5	
	Discharge	The main symptoms, signs, and auxiliary examination results are clearly and completely recorded.	2	
	Discharge diagnosis	Comprehensive and accurate discharge diagnosis	2	
	Discharge Instructions	Comprehensive and specific (drug and non-drug treatment, life guidance, time for return visit)	2	
Total			100	

Examiner's signature:

Clinical Practice Ability Scoring Table

Scoring Table for Clinical Thinking and Decision-making

Candidate name		Candidate Number	
Training discipline		Training base	
Assessment base		Assessment time	
**Scoring items**	**Criteria**	**Standard score**	**Score**
1	Summary of medical history	15	
2	Diagnosis and evidence	15	
3	Key points of differential diagnosis	15	
4	Treatment principles and measures	15	
5	Answers to professional questions	15	
6	Answers to questions on ethics, humanities, and professional literacy	15	
7	Ability of conclusion and communication skills	10	
Total		100	

Examiner's signature:

Clinical Practice Ability Scoring Table

Cardiopulmonary Resuscitation

Candidate name		Candidate Number	
Training discipline		Training base	
Assessment base		Assessment time	

**Scoring items**	**Criteria**	**Standard score**	**Score**
Assessment	Assess environmental safety.	5	
Identify and call for help	Evaluate the patient's response, call for help, start the emergency system and acquire the defibrillator.	10	
Identify cardiac arrest	Check for breathing or breathing	5	
	Touch the carotid pulse with a duration between 5 seconds and 10 seconds.	5	
Position	Lie the patient on his or her back on a hard floor.<br>Stand on the right side of the patient.	5	
High-quality chest compressions	The compression site is the lower half of the sternum, which is slightly below the midpoint of the connection between the two nipples.	5	
	Cross hands and keep upper arms upright.	5	
	Perform chest compressions at a rate of 100 to 120 times/min.	5	
	Compression depth reaches 5-6 cm.	5	
	Is the chest wall fully rebound after each compression?	5	
	Compression interruption time does not exceed 10 seconds.	5	
Artificial respiration and chest compressions	Give the patient adequate ventilation with 2 artificial respirations after 30 compressions.	10	
Artificial respiration	The method ratio is correct (1 second for each blow and 2 blows for no more than 10 seconds)	10	
	Effective artificial respiration with lifted chest;<br>Avoidance of excessive ventilation	5	
Judgment of recovery effect	Check carotid pulse and spontaneous breathing after 5 cycles.	5	
Overall evaluation	Skilled operation process and correct order	10	
Total		100	

Examiner's signature:

Clinical Practice Ability Scoring Table

Tracheal Intubation

Candidate name		Candidate Number		
Training discipline		Training base		
Assessment base		Assessment time		
**Scoring items**	**Criteria**		**Standard score**	**Score**
Preparation	Wash hands, wear hats and masks, sign the informed consent.<br>Check the ID of the patient, assess the patient's status, and determine whether there is a possibility of difficult intubation (oral statement).		5	
	Choose a tracheal tube according to the patient's condition and check that the balloon is unobstructed and there is no leakage.<br>Prepare laryngoscope, choose lens, and check lights.		5	
	Others: gloves, sputum aspirator, balloon mask, tracheal intubation guide wire, syringe, tape, tooth pad, stethoscope		5	
Operation process	1. Place the patient on his or her back, tilt his or her head back, and raise his or her neck to keep the mouth, pharynx, and trachea in a straight line.<br>Oxygenation for 2 minutes through balloon mask (oral statement)		10	
	2. Push the right thumb away from the patient's lower lip and jaw and put the index finger against the incisors.<br>Use the opener, if necessary, to remove foreign bodies in the respiratory tract.		10	
	3. Hold the laryngoscope in the left hand and enter the oral cavity along the right corner.<br>Press the back of the tongue to reveal the uvula slowly.<br>Push the lens to the base of the tongue and lift the lens to reveal the glottis when seeing the epiglottis.		10	
	4. Inserted The tracheal tube along the groove of the tongue depressor to glottis under the laryngoscope, remove tracheal intubation guide wire and gently feed the endotracheal tube in a proper depth.<br>Place the dental pad, and pull out the laryngoscope (5-point deduction for tracheal intubation guide wire exceeding the tip of the tube, 5-point deduction for a second intubation, and no points for various failures).		15	
	5. Inflate 3 to 5 ml into the balloon at the tip of the catheter and use a simple artificial respirator to press in the gas.<br>Observe the thoracic ups and downs, and use a stethoscope to listen to the sounds of the two lungs for symmetry to determine whether the catheter is in the trachea (no points for failed insertion or fixation of tracheal tube).		15	
	6. Tape to fix tracheal tube and dental pad.		5	
Overall evaluation	Overall proficiency in the operation process		10	
	Q & A: Indications and contraindications for intubation		10	
Total			100	

Examiner's signature:

Clinical Practice Ability Scoring Table

Urinary Catheterization

Candidate name		Candidate Number	
Training discipline		Training base	
Assessment base		Assessment time	

**Scoring items**	**Criteria**	**Standard score**	**Score**
Judgment	Determine the operation according to the question (5 points for correct judgment, and no point for wrong judgment).<br>Start urinary catheterization after being informed by the examiner.	5	
Preparation	Check the ID of the patient.<br>Pay attention to protect the privacy of the patient.	3	
	Explain to the patient about the purpose and necessity of the operation.	3	
	Prepare items, wear masks and hats, and wash hands.	4	
	Items: vulva disinfection bag, urinary catheter bag, urinary catheter, drainage bag, syringe, gloves,<br>Physiological saline, paraffin oil, mid-single, etc.	5	
Operation process	1. Assist the patient to position and expose the vulva. Place the mid sheet under the patient's buttocks.	4	
	2. Initial disinfection of the vulva<br>(1) Open the vulva disinfection bag for initial disinfection on the treatment cart and place the curved plate (with tweezers and iodine cotton balls inside)<br>between the patient's legs (5 points).<br>Wear gloves with one hand, use the other hand to take the iodophor cotton ball through the tweezer, and disinfect the vulva in order. For man, sequentially disinfects the mons pubis, upper 1/3 of inner thigh, penis, and scrotum. Use gauze to push the foreskin backward, lift the penis, and sequentially disinfects the urethral opening, glans, and coronal groove. For woman, sequentially disinfects the mons pubis, upper 1/3 of inner thigh, labia majora, labia minora, urethral orifice, vaginal opening, and anus (10 points).<br>After sterilization is completed, put the curved plate and all the dirt into the packaging bag and move it to the lower level of the treatment vehicle (5 points).<br>Comments: ①10-point deduction for the wrong order; ②5-point deduction for using the same cotton ball when disinfecting different parts.	20	
	3. Sterilize hands again.	2	
	4. The second disinfection of the vulva<br>(1) Place the catheter bag between the patient's legs, open the bag under the principle of aseptic operation, pour iodine and physiological saline, and open the catheter, syringe, and drainage bag (5 points).<br>(2) Put on sterile gloves, take out the hole towel, and spread it on the patient's vulva, check and lubricate the catheter, and connect the catheter and drainage bag as needed (5 points).<br>(3) Disinfect in order. For male, use the gauze to lift the penis and sequentially disinfects the urethral opening, glans, coronal groove, and urethral opening (5 points). For female, separates the labia minora with the left hand and sequentially disinfects the urethral opening, minor labia, and	20	

Clinical Practice Ability Scoring Table

	urethral opening (5 points).<br>Comments: ①10-point deduction for the wrong order; ②5-point deduction for using the same cotton ball when disinfecting different parts		
	5. Catheterization<br>(1) For man, lift the penis 90°, insert the urinary catheter until seeing urine (20 ~ 22cm), and continue to insert 5 ~ 7cm. Inject 10 ～ 20ml water into the balloon and pull back the urinary catheter to fix it.<br>(2) For woman, separate the labia minora, insert the urinary catheter until seeing urine (4 ~ 6cm), and continue to insert 5 ~ 7cm. Inject 10 ～ 20ml water into the balloon and pull back the urinary catheter to fix it.<br>Comments: ①10-point deduction for inserting the the urinary catheter into the vagina in women; ②5-point deduction for unreplaced the foreskin in man; ③10-point deduction for no urine	15	
	6. Leave the specimen as needed, remove the hole towel, and hang the urine drainage bag at an appropriate height (the drainage bag position should be lower than the bladder).	4	
Post-processing	Place the patient's position and explain the patient's precautions.	5	
	Clean up and wash hands again.	2	
	Observe the patient's reaction and urine, write urinary catheterization records, and retain specimens for examination.	3	
Overall evaluation	Strong aseptic concept and appropriate human care	5	
	Complete the operation expertly within the required time.	5	
Questions	Answer 2 questions from the indications, contraindications, complications, and post-operation missions of this operation.	5	
Total		110	

Examiner's signature:

Clinical Practice Ability Scoring Table

Peritoneal puncture

Candidate name		Candidate Number		
Training discipline		Training base		
Assessment base		Assessment time		

**Scoring items**	**Criteria**	**Standard score**	**Score**
Judgment	Determine the operation according to the question (5 points for correct judgment, and no point for wrong judgment).<br>Start peritoneal puncture after being informed by the examiner.	5	
Preparation	Patient Preparation: Check patient ID (1 point). Evaluate the patient's condition, explain to the patient about the indications, and determine whether there are contraindications (1 point). Sign the informed consent form (2 points). Tell the patient to cooperate during the operation (1 point). Tell the patient to empty the bladder and assist the patients to position themselves: lying on their back (2 points). Do abdominal examinations (abdominal circumference, abdominal dullness), and measure blood pressure and pulse (1 point).	8	
	Prepare items, wear masks and hats, and wash hands.	2	
	Items: sterilized items, sterilized gloves, sterilized abdominal puncture bag, anaesthetics, gauze, tape, abdominal belt, etc. (3 points).<br>Check the date various disinfection items (2 points).	5	
Operation process	1. Position: Determine the puncture point according to the subject (usually the left Maier point), and if necessary, use B-ultrasound to help position.	15	
	2. Sterilize hands again and wear sterilized gloves.	5	
	3. Check anaesthetics (2% lidocaine), conduct anaesthesia layer by layer (5-point deduction for no drawback).	10	
	4. Check the patency and air tightness of the puncture needle. If the air tightness is good and there is no obstruction, clamp the needle tail latex tube with a haemostat (5 points). Fix the puncture skin with the one hand and puncture with the other hand (10 points). Connect a 50 ml syringe, loosen the haemostatic forceps, and slowly draw ascites (8 minutes). Clip the hose again (2 points). Remove the syringe and keep the specimen (5 points).<br>Comments: 5-point deduction for no penetrating and airtightness check for abdominal puncture needle; 5-point deduction for a large amount of ascites without moving needle and 10-point deduction for unsuccessful puncture.	30	
	5. Remove the puncture needle, disinfect the puncture area, and cover it with sterile applications.	5	
Post-processing	Evaluate the patient's vital signs again after the operation. Measure the abdominal circumference. Explain to the patient about the precautions. Oral statement of usage of abdominal belt after a large amount of fluid is drawn (1 point without oral description).	4	
	Sent for marked specimens for inspection (routine, biochemical examination, culture, etc.). Complete the operation record.	3	
	Clean up and wash hands again.	3	
Overall evaluations	Strong aseptic concept and appropriate human care	5	
	Complete the operation expertly within the required time.	5	

Clinical Practice Ability Scoring Table

Questions	Choose 2 questions from the indications, contraindications, complications, and post-operation missions of this operation	5	
Total		110	

Examiner's signature:

Clinical Practice Ability Scoring Table

Dressing

Candidate name		Candidate Number	
Training discipline		Training base	
Assessment base		Assessment time	

**Scoring items**	**Criteria**	**Standard score**	**Score**
Judgment	Determine the operation according to the question (5 points for correct judgment, and no point for wrong judgment).<br>Start peritoneal puncture after being informed by the examiner.	5	
Preparation	Check patient ID. Evaluate the incision situation, explain to the patient about the purpose, necessity, and complications.	5	
	Items: dressing packs, gauze, tapes, gloves, iodophors, saline, etc. (note the expiration date)	5	
	Wash hands, wear masks and hats, choose dressing sites to protect patients' privacy.	5	
Operation process	1. Place a medicine changing bowl and make sterile cotton balls.	5	
	2. Assist patients to position themselves and expose the dressing replacement site.	5	
	3. Remove the outer dressing by hand and place the contaminated dressing on the curved plate with the inner face up (5 points), and then gently remove the inner dressing with tweezers (5 points). If the secretions are dry and sticky, they can be moistened with physiological saline before being taken off.<br>Avoid damage to the granulation tissue and the new epithelium.	10	
	4. Evaluate the incision and the exudation.	5	
	5. Hold the tweezers to take cotton balls with both hands to clean the incision and replace the drainage (5 points). Distinguish 2 tweezers (5 points). Disinfect the incision and surrounding skin twice.<br>Correct order (5 points), correct disinfection range (5 points), no blank area (5 points).	25	
	6. Cover the incision and fix the dressing. Correct gauze matte surface (5 points), correct number of coverage layers (5 points), and correct length and position of the tape (5 points).	15	
Post-processing	Assess the patient's status, assist the patient in organizing clothing, and explain to the patient about the precautions.	5	
	Properly handle contaminated dressings.	5	
Overall evaluations	Strong aseptic concept and appropriate human care	5	
	Complete the operation expertly within the required time.	5	
Questions	Choose 2 questions from the principle of dressing for different incisions, precautions for operation and post-operation mission matters.	5	
Total		110	

Examiner's signature:

Clinical Practice Ability Scoring Table

Surgical Sterilization

Candidate name		Candidate Number	
Training discipline		Training base	
Assessment base		Assessment time	

Scoring items	Criteria	Standard score	Score
Judgment	Determine the operation according to the question (5 points for correct judgment, and no point for wrong judgment).<br>Start peritoneal puncture after being informed by the examiner.	5	
Preparation	Check patient information, surgical sites, and surgical name.	5	
	Wear masks and hats, and wash hands in a standardized way (oral statement).	5	
	The assistant opens the sterile surgical bag and counts the items. Prepare the disinfected bending disc and the cotton ball. Take toothless oval forceps.	5	
Operation process	1. Stand on the right side of the patient, disinfect the surgical field 3 times in a standardized way. Replace the cotton ball each time, and replace the oval for the last time.	5	
	2. Correct scope of disinfection according to the surgical requirements	5	
	3. Correct disinfection sequence	5	
	4. No blank for disinfection and no omission	5	
	5. After the disinfection is completed, the curved plate and oval forceps are placed outside the operating table.	2	
	6. Use 4 small sterile sheets, fold a little on one side of each piece (fold 1/4), lay one piece on each side of the incision, 1/4 face down (2 points). Towel spreading range is correct (3 points). The orders are tail side, opposite side, head side and the side close to the operator (5 points).	10	
	7. Use cloth pliers to fix the four corners.	3	
	8. Cooperate with the assistant to shop the order in a correct way. The head side exceeds the anaesthesia frame and the foot side exceeds the operating table.	5	
	9. Choose a a proper area to wear the surgical gown. Lift the two corners of the collar and gently shake the surgical gown to open it.<br>Do not face the outside of the clothing to yourself or touch other things.	5	
	10. Toss the surgical gown slightly upwards. Insert both hands into the sleeve of the gown at the same time with both arms stretched forward, not over the shoulder. The upper body leaned forward slightly. A nurse or assistant assists in putting on and putting on the back strap.	5	
	11. Wear gloves correctly.	5	
	12. Untie the waistband of the surgical gown, turn around, and tie the waistband to the left waist.	5	
	13. After spreading the sheet on both sides, expand up and cover the anaesthesia frame. Try not move the position and pay attention to protecting the gloves when opening.	5	
Post-processing	Inform anaesthesiologists and nurses to start skin incision (5 points). Check the patient information and name of operation again. Estimate operation time and	10	

Clinical Practice Ability Scoring Table

	bleeding volume (5 points).		
Overall evaluation	Fluent operation process	5	
	Strong aseptic concept and appropriate human care	5	
Questions	Choose 2 questions from abdominal surgery incision location, scope of disinfection, and precautions related to preoperative preparation (skin preparation, pre-treatment preventive antibiotics, etc.).	5	
Total		110	

Examiner's signature:

Clinical Practice Ability Scoring Table

Gastric Tube Insertion

Candidate name		Candidate Number	
Training discipline		Training base	
Assessment base		Assessment time	

**Scoring items**	**Criteria**	**Standard score**	**Score**
Judgment	Determine the operation according to the question (5 points for correct judgment, and no point for wrong judgment).<br>Start peritoneal puncture after being informed by the examiner.	5	
Preparation	Patient Preparation: Check patient ID (1 point). Evaluate the patient's condition, explain to the patient about the indications, and determine whether there are contraindications (3 points). Sign the informed consent form and tell the patient to cooperate during the operation (2 points). Assist the patients to position themselves: take a semi-recumbent or sitting position (2 points). Measure blood pressure and pulse (2 points).	10	
	Prepare items, wear masks and hats, and wash hands.	2	
	Items: flashlight, therapeutic towel, curved plate, sterile cotton swab, disposable pus basin, stethoscope, tape, gloves, gastric tube, 20 ml syringe, paraffin oil, dressing bowl filled with clean water, pins, gauze, etc. (1 point). Check the expiration date of various items (1 point). The items are placed on the right side of the operator (1 point).	3	
Operation process	1. Cover the patient with a therapeutic towel under the jaw and slightly curve the patient's neck. Put the bending disc in a proper place. For the comatose or poisoned patient, take the left side supine or supine position.	5	
	2. Ask about the medical history of the nasal cavity and check the nasal cavity. Select the side of the nasal cavity with smooth ventilation and clean the nasal cavity with a cotton swab.	5	
	3. Check the gastric tube, measure the insertion length of the gastric tube, and make a mark. The adult is generally about 55 cm.	10	
	4. Lubricate the gastric tube and close the distal end of the gastric tube.	10	
	5. Gradually insert the gastric tube to 55cm, and confirm that there is no curling in mouth during the insertion process.	15	
	6. Judge whether the gastric tube is in the stomach (no score for inserting into the trachea or having curling in the mouth)	10	
	7. Fix it to the nose with tape.	5	
	8. When indwelling is required, fold back the end of the gastric tube and wrap it with gauze, and fixed beside the patient.	5	
Post-processing	Assess the patient's status, assist the position, and explain to the patient about the precautions.	5	
	Properly handle items by putting contaminated items into disposable pus basin.	5	
Overall evaluation	Strong aseptic concept and appropriate human care	5	
	Complete the operation expertly within the required time.	5	
Questions	Choose 2 questions from the indications, contraindications, complications, and post-operation missions of this operation.	5	
Total		110	

Examiner's signature:

Clinical Practice Ability Scoring Table

Thoracentesis

Candidate name		Candidate Number	
Training discipline		Training base	
Assessment base		Assessment time	

Scoring items	Criteria	Standard score	Score
Judgment	Determine the operation according to the question (5 points for correct judgment, and no point for wrong judgment).<br>Start peritoneal puncture after being informed by the examiner.	5	
Preparation	Patient Preparation: Check patient ID (1 point). Evaluate the patient's condition, explain to the patient about the indications, and determine whether there are contraindications (1 point). Sign the informed consent form and tell the patient to cooperate during the operation (2 points).	5	
	Prepare items, wear masks and hats, and wash hands.	2	
	Items: disinfection items, sterile gloves, sterile chest puncture bag, anaesthesia, rescue items (0.1% epinephrine and syringe), applicator, stethoscope, several sterile test tubes, etc. (4 points). Check the disinfection date of various disinfection items (4 points)	8	
Operation process	1. Tell the patient to sit on the backrest chair in reverse, with both arms lying flat on the upper edge of the chair back and the head lying on the forearm. If it is a severely ill patient, take a semi-recumbent position on the bed and raise his/her hand on the unhealthy side. Put a pillow under his/her head, or stretch the hand over the top of the head to expand the intercostal.	5	
	2. Locate the puncture point through physical examination (4 points). Usually, the puncture site is in the 7th to 8th intercostal space of the subscapular angle line or 6th to 7th intercostal space of the midaxillary line or the 5th intercostal space of the anterior axillary line (4 points). In the case of wrapped effusion, the puncture site should be determined based on X-ray or ultrasound findings (2 points).	10	
	3. Disinfect the puncture sites twice with a diameter about 15cm and no blank (3 points). Open chest wearers wear sterile gloves and drape (3 points). check the patency and air tightness of the chest needle sex (3 points); Check the anaesthetic (2% lidocaine, 2~5ml) and slowly puncture it vertically at the upper edge of the lower rib at the positioning point with a cycle of withdraw and injection (4 points). Infiltrate the puncture site layer by layer until inserting into the parietal pleura and seeing pleural effusion. Inject some anaesthetics (2 points).	15	
	4. Estimate the penetration depth of the puncture needle according to the anaesthesia injection needle (2 points). Clamp the latex tube of the needle tail with a haemostat (4 points). Fix the skin at the puncture site with the left index finger and middle finger. Slowly and vertically insert the puncture needle along the next ribs in the right hand. When passing through the parietal pleura, the needle tip resistance suddenly disappears and stops (4 points). Connect the syringe, release the haemostat, and draw back to see the pleural effusion (2 points). Tell the assistant to use haemostatic forceps to help close the latex tube in time (2 points). Inject the extracted liquid into the test tube and special container, and send the pleural effusion for inspection (1 point).<br>Comment: 10-point deduction for failed thoracentesis for 2 times.	15	

Clinical Practice Ability Scoring Table

	5. After pumping, tell the patient to hold the breath when pulling out the puncture needle (3 points). Cover the puncture site with sterile gauze, press it for a while, and fix the tape (2 points). After the operation, tell the patient to take a rest in bed and observe whether there are uncomfortable responses (2 points). After pumping, conduct routine auscultation and pay attention to breathing sounds. Conduct X-ray examination if necessary (3 points).	10	
	6. (Oral statement) Do not pump too much fluid or too fast to prevent the occurrence of reexpansive pulmonary edema (5 points). For the purpose of diagnosis, draw 50～100 ml. For the purpose of decompression, the first time should not exceed 600 ml and not exceed 1000 ml (5 points) each time.	10	
Post-processing	Position the patient, reassess the patient's vital signs, assess the breathing situation, and explain to the patient about the precautions.	4	
	Sent pleural effusion for inspection (routine, biochemical examination, culture)	3	
	Properly handle items by putting contaminated items into disposable pus basin.	3	
Overall evaluation	Strong aseptic concept and appropriate human care	5	
	Complete the operation expertly within the required time.	5	
Questions	Choose 2 questions from the indications, contraindications, complications, and post-operation missions of this operation.	5	
Total		110	

Examiner's signature:

Clinical Practice Ability Scoring Table

Lumbar Puncture

Candidate name		Candidate Number	
Training discipline		Training base	
Assessment base		Assessment time	

**Scoring items**	**Criteria**	**Standard score**	**Score**
Judgment	Determine the operation according to the question (5 points for correct judgment, and no point for wrong judgment).<br>Start peritoneal puncture after being informed by the examiner.	5	
Preparation	Patient Preparation: Check patient ID (1 point). Evaluate the patient's condition, explain to the patient about the indications, and determine whether there are contraindications (1 point). Sign the informed consent form and tell the patient to cooperate during the operation (2 points).	5	
	Prepare items, wear masks and hats, and wash hands.	2	
	Items: sterilized items, sterile gloves, lumbar puncture bags, anaesthetics, syringes, gauze, tape, etc. (4 points). Check the date of various disinfection items (4 points)	8	
Operation process	1. Take the left side position or the right side position according to the condition. Assist the patient to bend hips and knees to hold head. Make the person comfortable so that they can tolerate a longer operation time	5	
	2. Select a suitable puncture point. Mark the puncture point which is the intersection between the highest point of the iliac crest and the posterior midline (equivalent to the first 3~4 lumbar spinous process space). It can also be performed in the previous or next lumbar space	5	
	3. Disinfect puncture point twice with a range of about 15 cm in diameter (no point for lager area at the second time of disinfection or leaving blank).	5	
	4. Wear sterile gloves	2	
	5. Open the puncture bag and check the equipment. Check whether the puncture bag is within the valid period of disinfection, and check whether the items in the bag are complete, whether the puncture needle is unobstructed.	5	
	6. Spread sterile hole towel.	3	
	7. Check anaesthetics, draw 5 ml of 2% lidocaine, and perform local infiltration anaesthesia from the skin to the intervertebral ligament at the puncture point (5-point deduction for unchecked anaesthetics).	5	
	8. Fix the skin at the puncture point with one hand, and slowly pierce the puncture needle with the other hand in the direction perpendicular to the back. Needle tip slope must be upward. Slowly pull out the needle core when feeling 2 breakthroughs, and see the cerebrospinal fluid flowing out. Adults generally have a needle penetration depth of 4 to 6 cm. Pay attention to observe the patient's reaction during the puncture (3-point deduction for not putting the needle tip slope level upwards, 3-point deduction for a failed puncture, and 10-point deduction for fails more than 3 times).	20	
	9. Measure intracranial pressure, and press the neck and abdomen to test pressure.	10	
	10. Pull back the needle core, pull out the puncture needle, cover the sterile gauze, disinfect the puncture point, cover with gauze, and fix the tape.	5	
	Position the patient, reassess the patient's vital signs, and explain to the patient about the precautions such as lying on bed without pillows for 4-6 hours.	5	

Clinical Practice Ability Scoring Table

Lumbar Puncture

Post-processing	Sent cerebrospinal fluid for inspection (routine, biochemical examination, culture, etc.)	3	
	Properly handle items by putting contaminated items into disposable pus basin.	2	
Overall evaluation	Strong aseptic concept and appropriate human care	5	
	Complete the operation expertly within the required time.	5	
Questions	Choose 2 questions from the indications, contraindications, complications, and post-operation missions of this operation.	5	
Total		110	

Examiner's signature:

Clinical Practice Ability Scoring Table

ECG Operation

Candidate name		Candidate Number	
Training discipline		Training base	
Assessment base		Assessment time	

Scoring items	Criteria	Standard score	Score
Judgment	Determine the operation according to the question (5 points for correct judgment, and no point for wrong judgment).<br>Start peritoneal puncture after being informed by the examiner.	5	
Preparation	Wear neat clothes and wash hands.	2	
	Items: electrocardiograph, lead wire, electrical plug-in board (preparation), treatment plate, alcohol cotton ball, container for dirt, treatment vehicle	5	
	Close doors and windows, set screens when necessary, and leave family and accompany guests.	3	
	Check the patient ID, explain the purpose, and assist the position (lying on bed).	5	
Operation process	1. Expose the inside of both wrists, the medial ankles of both lower extremities. Loosen the clothes button.	5	
	2. Wipe the lead connection with alcohol cotton ball.	5	
	3. Connect the lead wire correctly (red for right arm, yellow for left arm, green for left leg and black for right leg. (16 points, 4-point deduction for each wrong connection). V1 (red): 4th intercostal space on the right sternal border (4 points); V2 (yellow): 4th intercostal space on the left sternal border (4 points); V3 (green)<br>:mid-poin to the connection between V2 and V4 (4 points);V 4(brown): the intersection of the left midclavicular line and the fifth intercostal space (4 points); V5 (black): the anterior axillary line at the same level as V4 (4 points); V6 (purple): At the mid-axillary line at the same level as V4 (4 points).	40	
	4. Adjust parameters with an oral statement of calibration voltage and tracing speed.	5	
	5. Trace the electrocardiogram with 3 complete waveforms.	5	
	6. Remove the lead wires and shut down.	5	
Post-processing	Place the patient and arrange items.	5	
	Mark the name, date, time, and ECG lead on the ECG and check if there is any change.	5	
Overall evaluation	Kind attitude with good human care	5	
	Complete the operation expertly within the required time.	5	
Questions	Read 2 ECGs on site	5	
Total		110	

Examiner's signature:

Clinical Practice Ability Scoring Table

Superficial Wound Debridement

Candidate name		Candidate Number		
Training discipline		Training base		
Assessment base		Assessment time		

**Scoring items**	**Criteria**	**Standard score**	**Score**
Judgment	Determine the operation according to the question (5 points for correct judgment, and no point for wrong judgment).<br>Start peritoneal puncture after being informed by the examiner.	5	
Preparation	Patient Preparation: Check patient ID (1 point). Evaluate the patient's condition, explain to the patient about the indications, and determine whether there are contraindications (1 point). Sign the informed consent form and tell the patient to cooperate during the operation (2 points).	5	
	Prepare items, wear masks and hats, and wash hands.	2	
	Items: debridement bag, sterile dressing, sterile gloves, scissors or razor for skin preparation, bandage, tape, saline, 3% hydrogen peroxide solution, iodophor, 2% lidocaine injection, 10 ml syringe, brushes, instrument tables, trays and tray racks, needle barrels, dirt container (4 points). Check the date of various disinfection items (4 points).	8	
Operation process	1. Open the debridement bag and wear sterile gloves.	2	
	2. Cover the wound with sterile gauze. If there is hair around the wound, shave it first.If there is oil, wipe it off with alcohol.	5	
	3. Scrub the skin around the wound twice with water and with a soft brush dipped in soapy liquid, and rinse the skin around the wound with physiological saline to remove dirt. Be careful not to allow flushing fluid to flow inside the wound.	5	
	4. Remove the gauze covering the wound, rinse the wound with sterile saline, and use sterile small.<br>Use gauze to gently wipe off dirt and foreign bodies in the wound, rinse the wound with 3% hydrogen peroxide solution. Rinse the wound with sterile saline twice after the wound surface appears foam.	5	
	5. Dry the skin, disinfect the surrounding of the wound with iodophor, replace the sterile gloves, and spread the sterile small hole towel.	5	
	6. Oral statement of local infiltration anaesthesia procedure, anaesthetics, injection point, level and orders.Withdraw before injection.	5	
	7. Remove skin and tissue that have been inactivated due to tears and bruises. Remove foreign objects, blood clots, etc.	5	
	8. After thorough debridement, flush the wound again with sterile saline and 3% hydrogen peroxide solution. Dry the skin	5	
	9. Replace instruments, replace sterile gloves, and then lay a layer of sterile small hole towel.	5	

Clinical Practice Ability Scoring Table

Superficial Wound Debridement

	10. Thread the needle after pinching the needle. After inserting the needle, press the eye of the needle with your thumb and pinch out the thread with your index finger. Align the needle at the junction of the tail 1/3 and the needle tip 2/3; align the needle holder to the head. Pinch the needle eye and thread with the left thumb and index finger, with the needle tip facing forward and the needle holder holding palms up, needle holder against left thumb, index finger glove. Clamp the needle.	5	
Operation process	11. Use left hand to hold the short side of the forceps to fix or clamp the tissue to be sutured. Use the right hand to hold the needle holder along the arc of the needle to pierce the needle (vertically into the needle), and penetrate the tissue (vertically out of the needle). Fix the needle tip with tweezers, use the needle holder to pull the needle out of the tissue, place the tweezers in the left palm, pinch the needle eye and the thread with the left thumb and index finger, pull the thread, and finish knotting (no point for fake knots or slip knots).	5	
	12. Suture in layers using the method of simple intermittent suture to avoid embedding other tissues. The stitching should be tight.	5	
	13. The margin of the needle on both sides of the incision is about 0.5 cm. The needle distance is about 1.0 cm. the depth is suitable. The incision is well aligned and the ligation is tight.	5	
	14. After stitching, disinfect the skin and wrap it with gauze.	3	
Post-processing	Evaluate the patient again after the operation, assist the position, and inform the precautions (injection of tetanus needle)	5	
	Properly handle items by putting contaminated items into disposable pus basin.	5	
Overall evaluation	Strong aseptic concept and appropriate human care	5	
	Complete the operation expertly within the required time.	5	
Questions	Choose 2 questions from different wound debridement timing, treatment principles, and tetanus prevention principles.	5	
t otal		110	

Examiner's signature:

Clinical Practice Ability Scoring Table

Bone Marrow Aspiration

Candidate name		Candidate Number	
Training discipline		Training base	
Assessment base		Assessment time	

Scoring items	Criteria	Standard score	Score
Judgment	Determine the operation according to the question (5 points for correct judgment, and no point for wrong judgment).<br>Start peritoneal puncture after being informed by the examiner.	5	
Preparation	Patient Preparation: Check patient ID (1 point). Evaluate the patient's condition, explain to the patient about the indications, and determine whether there are contraindications (1 point). Sign the informed consent form and tell the patient to cooperate during the operation (2 points).	8	
	Prepare items, wear masks and hats, and wash hands.	2	
	Items: sterilized items, sterile gloves, bone piercings, slides, anaesthetics, syringes, sterile gauze, etc., check the sterilization date of various sterilized items.	5	
Operation process	Choose the proper posture according to the condition. Posterior position for thoracic spine and anterior superior iliac spine puncture; Lateral position for supine iliac posterior superior iliac spine; Sitting or lateral position for spinous process puncture (1 point each).<br>Anterior superior iliac spine: 1 ～ 2 cm behind anterior superior iliac spine.<br>Posterior superior iliac spine: both sides of the sacral spine, protruding parts above the hips.	5	
	2. Disinfect area skin twice at a diameter of 15 cm, wear sterile gloves, and lay sterile hole towel.	5	
	3. Check the instruments. Pay attention to whether the puncture needle and the syringe are dry, whether the syringe nipple coincides with the puncture needle, whether the needle core and the puncture needle are matched, and whether the needle is sharp (2 points). Adjust puncture depth, 1.0 cm for sternum puncture and 1.5 cm for iliac puncture (3 points).	5	
	4. Check the anaesthetic (2 points). Perform local anaesthesia with 2% lidocaine, with orange peel-like cumulus changes at the skin at the first subcutaneous injection (4 points). Insert the needle vertically and perform layered anaesthesia to periosteum.<br>Note: draw back before pushing the anaesthetic to see if it is in the blood vessel (4 points).	10	
	5. Use the left thumb and index finger to fix the puncture site, and the right hand to hold the bone marrow puncture needle. If it is a sternum puncture, it should be inserted at an angle of 30° -40° with the bone surface (5 points). After the tip of the puncture needle touches the bone, rotate the puncture needle left and right along the long axis of the needle body of the puncture needle, and adjust it forward to slowly to penetrate the bone (5 points). When the puncture resistance suddenly disappeared and the puncture needle was fixed in the bone, which indicates that the puncture needle had entered the bone marrow cavity (5 points). If the puncture needle has not been fixed, it	20	

Clinical Practice Ability Scoring Table

	should continue to puncture a little to a fixed point (5 points).		
	6. Pull out the core of the puncture needle, connect a dry syringe, and draw the bone marrow fluid with appropriate strength. The extracted bone marrow fluid should generally be 0.1 ～ 0.2ml	5	
	7.Drop the bone marrow on the glass slide, immediately push the slide to prepare several smears of bone marrow fluid. Correct smear method Place the slide at a 30 ° angle, drop the sucked bone marrow on the top of the slide, hold a clean slide horizontally in the left hand, dip a small amount of bone marrow tissue with the tip of the other slide in the right hand, and drop it on The top of the left hand slide,<br>Use the right hand slide edge to smear horizontally. No bone marrow fluid is drawn without score	10	
	8. After extracting the bone marrow fluid, reinsert the needle core. Pull out the puncture needle and cover the sterile gauze (the needle core is not inserted for 5 minutes)	5	
	Total	110	

Examiner's signature:

Clinical Practice Ability Scoring Table

Put on and Take off A Gown

Candidate name		Candidate Number	
Training discipline		Training base	
Assessment base		Assessment time	

**Scoring items**		**Criteria**	**Standard score**	**Score**
Judgment		Determine the operation according to the question (5 points for correct judgment, and no point for wrong judgment). Start peritoneal puncture after being informed by the examiner.	5	
Preparation		Wear clean clothes, roll sleeves over elbows, trim nails, and remove watches.	4	
		Wash hands, wear masks and hats	5	
		Choose a clean and spacious area, and pay attention to isolation requirements.	2	
		Choose a suitable gown.	4	
Operation process	Wear a gown	Take a gown and with the clean side towards yourself. Check whether the tie is contaminated.	4	
		Check isolation clothing (size, damage, pollution).	4	
		Wear sleeves correctly (note whether the sleeves touch the face and mask).	6	
		No pollution when tie neckline	5	
		Tie cuffs.	4	
		Fold the edge of the isolation garment correctly (check whether the inner garment is completely covered, and check whether the inner surface of the isolation garment is exposed, check whether the hem of the isolation garment is folded in and fully covered).	6	
		The method of tying the belt is correct.	6	
	Take off the gown	Loose belts and tie knots.	2	
		Loose cuffs, folded sleeves (over elbow).	5	
		Disinfect hands and forearms.	5	
		Untie the neckline and do not contaminate the tie.	5	
		The method of undressing sleeves is correct (note the contamination of forearms).	8	
		Organize the isolation clothing, hang it correctly, and expose the non-contaminated surface (when hanging in the clean area)	5	
Post-processing		Wash hands again.	5	
		The face and mask cannot touch the outside of the gown.	5	
Overall evaluation		Strong aseptic concept (5 points) Complete the operation expertly within the required time.	10	
Questions		Answer the scope of use of the gown. Point out the contaminated area and clean area of the used gown. How to hang it if the worn gown needs to be re-worn?	5	
Total			110	

Examiner's signature:

Clinical Practice Ability Scoring Table

Sputum Suction Operation

Candidate name		Candidate Number		
Training discipline		Training base		
Assessment base		Assessment time		

Scoring items	Criteria	Standard score	Score
Judgment	Determine the operation according to the question (5 points for correct judgment, and no point for wrong judgment).<br>Start peritoneal puncture after being informed by the examiner.	5	
Preparation	Check medical orders, patient ID (1-point deduction for each missing item)	3	
	Evaluate the patient's condition, sputum volume and sputum viscosity (auscultation), vital signs, respiratory status, sputum sound, SpO2, psychology, cooperation, mouth, nasal mucosa; those with gastric tube feeding, before sputum suction Suspension of tube feeding (2-point deduction for each incomplete assessment and non-auscultation).	3	
	Inform the patient about the operation purpose, risks and discomforts, and tell the awake patients to cooperate (immediate implementation of sputum suction when the phlegm is critical, 1 point will be deducted for each missing item until the deduction is completed).	3	
	Wash hands, wear masks and hats, and provide occupational protection when necessary (1 point for each missing item).	3	
	Items: negative pressure suction device, 2 suitable suction tubes, treatment tray, treatment towel, sterile treatment, 2 treatment bowls, 1 sterile forceps, gloves, sterile saline, glass joint and stethoscope. Prepare tongue depressor, mouth opener, tongue pliers, flashlight, etc. if necessary, and place them reasonably. Connect and check the performance of the sputum suction device, and adjust the negative pressure (2 points deducted for incomplete items)	3	
Operation process	1. Assist the patient to take a proper position, check the oral mucosa, and remove the active denture (oral)	5	
	2. Observe the operating regulations. Connect the sputum suction device correctly and safely	5	
	3. Wear gloves. Connect the sputum suction tube, test suction, and moisten the catheter (2-point deduction for no test suction or moistening the catheter).	10	
	4. Do not give negative pressure when inserting the suction tube, gently put the suction tube in a suitable position. Withdraw 1cm when giving negative pressure in case of resistance (2-point deduction for inserting too deep or too shallow).	10	
	5. Rotate the tube in left and right directions, lift upward, and suck the sputum (no more than 15 seconds each time, with an interval of 3 to 5 minutes).<br>Comment: 3-point deduction each for long suction time, violent action and incorrect orders).	10	
	6. Keep the suction hose flush at intervals, take off the glove from the inside of the glove and wrap the suction tube and discard the unwashed (3-point deduction for sputum blockage).	10	
	7. Observe the improvement of the patient's respiratory tract, sputum traits, auscultate whether the lung wet rales reduce or disappear, and observe the vital signs, SpO2.	10	
	8. Record sputum volume, traits, color.	5	
Post-processing	Assist the patient to position and explain the precautions to patient.	3	
	Restore the instruments.	3	
	Proper waste dealing	4	

Clinical Practice Ability Scoring Table

Overall evaluation	Pay attention to intraoperative evaluation, care about patients, and strictly follow aseptic procedures (5 points).<br>Proficient and accurate operation, correct sputum suction method and a timely completion (5 points).	10	
Questions	Choose 2 questions from the indications, contraindications, complications, and post-operation missions of this operation.	5	
Total		110	

Examiner's signature:
